# An Update of the Evolving Epidemic of *bla*_KPC_ Carrying *Klebsiella pneumoniae* in Sicily, Italy, 2014: Emergence of Multiple Non-ST258 Clones

**DOI:** 10.1371/journal.pone.0132936

**Published:** 2015-07-15

**Authors:** Celestino Bonura, Mario Giuffrè, Aurora Aleo, Teresa Fasciana, Francesca Di Bernardo, Tomaso Stampone, Anna Giammanco, The MDR-GN Working Group, Daniela Maria Palma, Caterina Mammina

**Affiliations:** 1 Department of Sciences for Health Promotion and Mother-Child Care “G. D’Alessandro”, University of Palermo, Palermo, Italy; 2 Laboratory of Microbiology, ARNAS “Civico, Di Cristina and Benfratelli”, Palermo, Italy; 3 Laboratory of Microbiology, Azienda Ospedaliera Ospedali Riuniti “Villa Sofia-V. Cervello”, Palermo, Italy; 4 Laboratory of Microbiology, Azienda Ospedaliero-Universitaria”Paolo Giaccone”, Palermo, Italy; 5 II Intensive Care Unit, ARNAS “Civico, Di Cristina and Benfratelli”, Palermo, Italy; St. Petersburg Pasteur Institute, RUSSIAN FEDERATION

## Abstract

**Background:**

In Italy, *Klebsiella pneumoniae* carbapenemase producing *K*. *pneumoniae* (KPC-Kp) strains are highly endemic and KPC producing CC258 is reported as the widely predominating clone. In Palermo, Italy, previous reports have confirmed this pattern. However, recent preliminary findings suggest that an epidemiological change is likely ongoing towards a polyclonal KPC-Kp spread. Here we present the results of molecular typing of 94 carbapenem non susceptible *K*. *pneumoniae* isolates detected during 2014 in the three different hospitals in Palermo, Italy.

**Methods and Results:**

Ninety-four consecutive, non replicate carbapenem non susceptible isolates were identified in the three largest acute general hospitals in Palermo, Italy, in the six-month period March-August 2014. They were characterized by PCR for β-lactam, aminoglycoside and plasmid mediated fluoroquinolone resistance genetic determinants. The *mgrB* gene of the colistin resistant isolates was amplified and sequenced. Clonality was assessed by pulsed field gel electrophoresis and multilocus sequence typing. Eight non-CC258 sequence types (STs) were identified accounting for 60% of isolates. In particular, ST307 and ST273 accounted for 29% and 18% of isolates. CC258 isolates were more frequently susceptible to gentamicin and non-CC258 isolates to amikacin. Colistin non susceptibility was found in 42% of isolates. Modifications of *mgrB* were found in 32 isolates.

**Conclusions:**

Concurrent clonal expansion of some STs and lateral transmission of genetic resistance determinants are likely producing a thorough change of the KPC-Kp epidemiology in Palermo, Italy. In our setting *mgrB* inactivation proved to substantially contribute to colistin resistance. Our findings suggest the need to continuously monitor the KPC-Kp epidemiology and to assess by a nationwide survey the possible shifting towards a polyclonal epidemic.

## Introduction

In Italy, *Klebsiella pneumoniae* carbapenemase producing *K*. *pneumoniae* (KPC-Kp) strains are highly endemic [1]. After the first report in 2008 [2], KPC-Kp have widely spread, and several healthcare-associated outbreaks have been described [1]. The significantly increasing trend for carbapenem resistance in *K*. *pneumoniae* in Italy has been recently confirmed by the 2013 surveillance report of the European Antimicrobial Resistance Surveillance Network (EARS-Net). In this country, indeed, the mean prevalence of carbapenem resistant *K*. *pneumoniae* has risen from 15.2% to 34.3% in 2013 [3]. A rapid dissemination of colistin-resistant isolates of KPC-Kp on a national scale has been recently reported [4]. The pandemic high-risk clonal complex (CC) 258 has so far massively contributed to the Italian epidemic spread of KPC-Kp, with only a marginal role for other lineages or strains carrying different resistance mechanisms [4].

In 2008, the II intensive care unit (ICU) of the acute general hospital "ARNAS Civico, di Cristina e Benfratelli” (ARNAS GH), Palermo, Italy, was affected by the first outbreak of KPC-3 producing *K*. *pneumoniae* ST258 occurring in Sicily [5]. Since then, colonization and infection with this hyperendemic clone have become a key epidemiological feature of many healthcare facilities in this region [6–7]. Spread of colistin-resistant *K*. *pneumoniae* has been also described [8,9]. In particular, an outbreak involving different wards of the ARNAS general hospital Civico, di Cristina e Benfratelli in Palermo, Italy, was reported in the period from June to December 2011 [9].

In March 2014, while performing a program of active surveillance cultures (ASCs) in five neonatal intensive care units (NICUs) of Palermo, Italy, three isolates of KPC-Kp were identified from three infants admitted to two different NICUs. Molecular typing showed that the three KPC-Kp isolates belonged to two different STs, namely ST307 and ST323. Following this identification, all the carbapenem non susceptible isolates identified in April and May 2014 in the ARNAS GH and a second large acute general hospital, the “Azienda Ospedaliera Villa Sofia-V. Cervello” (AO VSC) were collected and submitted to typing. This preliminary study found that out of 16 isolates seven belonged to ST307, six to ST258 and three to ST273 [10].

Here we present the results of molecular typing of the carbapenem non susceptible *K*. *pneumoniae* isolates detected in the six month-period March-August 2014 in the three largest acute general hospitals, i.e ARNAS GH, AO VSC and the “Azienda Ospedaliero-Universitaria Policlinico P. Giaccone” (AOUP) of Palermo, Italy. The high prevalence of colistin resistance in our epidemiological setting prompted also us to confirm the role of possible modifications in the *mgrB* gene based upon the findings of recent studies identifying this gene as a critical target for the development of colistin resistance in *K*. *pneumoniae* [11,12].

## Materials and Methods

### Isolates

During the period from 1^st^ March to 31 August 2014, the microbiology laboratories of the three enrolled hospitals collected all the consecutive non-replicate clinical isolates of *K*. *pneumoniae* from any site of infection or colonization, that exhibited minimum inhibitory concentrations (MICs) for imipenem and/or meropenem higher than 1 mg/L. All isolates were then transferred to the molecular epidemiology laboratories of AOUP for confirmation of species identification and carbapenem MICs, characterisation of the carbapenem resistance genetic determinants and analysis of clonality. Information on the source of isolates and type of ward was also recorded.

### Characterization of isolates

Bacterial identification and antimicrobial susceptibility testing had been carried out by the collecting laboratories using either the Phoenix Automated Microbiology System (Becton Dickinson Diagnostic Systems, Sparks, United States) or the Vitek-2 System (bioMérieux, Marcy l’Etoile, France).

Confirmatory MIC testing for imipenem and meropenem was carried out by Etest (bioMérieux). All isolates confirmed to be non-susceptible to imipenem and/or meropenem according to the (European Committee on Antimicrobial Susceptibility Testing (EUCAST) breakpoints [13] were further analyzed. MICs for colistin were determined by Etest (bioMérieux).

KPC-Kp were first evaluated for carbapenemase production by meropenem plus EDTA and meropenem plus phenylboronic acid using the disk diffusion method [14]. Polymerase chain reaction (PCR) screening for carbapenemase encoding genes of classes A (*bla*_KPC_, *bla*_GES_), B (*bla*_VIM_, *bla*_IMP_, *bla*_NDM_) and D (*bla*_OXA-48_), for extended-spectrum β-lactamases encoding genes *bla*_TEM_, *bla*_OXA_, *bla*_SHV_ and *bla*_CTX-M_ and for plasmid-mediated quinolone resistance (PMQR) genes *qnr*A, *qnr*B and *aac(6’)-Ib-cr* was performed [15,16].The identity of resistance genes was confirmed by DNA sequence analysis. Because direct sequencing has been proved to be not sufficiently discriminating, further analysis was carried out by PCR and sequencing to identify *bla*_CTX-M-15_ [17]. The *mgrB* locus of colistin resistant isolates was amplified by PCR using primers mgrB_Ext_F and mgrB_Ext_R, as previously published, and sequenced [11].

### Analysis of clonality

All KPC-Kp isolates were submitted to pulsed-field gel electrophoresis (PFGE) of genomic DNA after digestion with *Xba*I with a CHEF Gene Mapper system (Bio-Rad Laboratories, Kingdom), and results interpreted using previously described criteria [18]. Isolates with indistinguishable profiles were attributed with a pulsotype named by a capital letter (A to H). Subtypes were identified and named by the same capital letter followed by an Arabic number (e.g. A1) when the profiles of isolates differed by one to four bands. PFGE profile of ST258 isolates was identified by a visual comparison with previously characterized isolates [5] and labeled with the letter O (old) irrespective of band differences.

Multilocus sequence typing (MLST) of a total of 15 KPC-Kp isolates representative of each PFGE type/subtype, except for only two randomly selected isolates belonging to pulsotype O, was performed and STs were assigned using the MLST web site [http://www.pasteur.fr/recherche/genopole/ PF8/mlst/Kpneumoniae.html].

## Results

During the study period a total of 94 consecutive non-replicate, carbapenem non susceptible isolates were identified in the three hospitals participating in the study.

Fifty-seven out of 94 isolates (61%) were isolated from ICU patients, whilst the remaining were from medical wards, nephrology and renal transplant units and long term care units. Three isolates came from two different NICUs where active surveillance culture programs were working [10]. Clinical sources were as follows: 34 isolates were from urine, 27 from blood, 16 from bronchial aspirates, 10 from wound swabs or drainages, 7 from feces or rectal swabs.

All the 94 isolates had imipenem and/or meropenem MICs ranging between 8 and ≥16 μg/mL (Table 1). The proportions of isolates non susceptible to amikacin, gentamicin and colistin were, respectively, 40% (38 isolates), 49% (46 isolates) and 42% (39 isolates). Only one isolate proved to be susceptible to ciprofloxacin.

**Table 1:** Main phenotypic and genetic characteristics of 94 carbapenem non susceptible KPC-Kp isolates, Palermo, Italy, 2014. ST, sequence type; MICs, minimum inhibitory concentrations; IPM, imipenem; MEM, meropenem; AMK, amikacin; GEN, gentamicin; COL, colistin.

ST	Nr. isolates	Hospital^*^	Pulsotype/ subtype	Resistance genetic determinants				MICs (μg/mL)				
				*KPC*	*CTX-M-15*	*aac6-Ib-cr*	*qnrB*	IPM	MEM	AMK	GEN	COL†
258	21	A	O	+	-	+	-	8 - ≥16	≥16	16 - ≥64	≤1–8	≤0.5 - >256
	9	B	O	+	-	+	-	8 - ≥16	≥16	16 - ≥64	≤1–2	≤0.5 - >256
	7	C	O	+	-	+	-	8 - ≥16	≥16	16 - ≥64	≤1–2	≤0.5 - >256
512	1	B	I	+	-	+	-	≥16	≥16	≥64	≤1	≤0.5
	8	C	D1	+	+	+	+	≥16	≥16	≤2–8	2 - ≥16	≤0.5–48
307	7	A	D1	+	+	+	+	8 - ≥16	8 - ≥16	≤2–8	2 - ≥16	48
	5	A	D1	+	-	+	+	8 - ≥16	8 - ≥16	≤2	≥16	≤0.5
	3	A	D2	+	+	+	+	≥16	≥16	4 - ≥64	2 - ≥16	3
	2	B	D1	+	+	+	+	≥16	≥16	≤2	≥16	≤0.5
	1	A	D3	+	-	+	+	≥16	≥16	≤2	≤1	≤0.5
	1	A	D4	+	-	+	+	≥16	≥16	≤2	≤1	≤0.5
273	11	A	C1	+	-	-	-	8 - ≥16	8 - ≥16	≤2	≥16	≤0.5–4
	2	A	C2	+	-	-	-	≥16	≥16	≥16	≥16	≤0.5
	2	B	C1	+	-	-	-	≥16	≥16	≤2	≥16	3–4
	2	A	C1	+	+	+	+	≥16	≥16	16	≥16	4
405	2	A	G	+	+	+	+	≥16	≥16	≤2	≥16	≤0.5
	1	B	G	+	+	+	+	≥16	≥16	≤2	≥16	≤0.5
	1	C	G	+	+	+	+	≥16	≥16	≤2	≥16	≤0.5
101	4	A	A	+	-	-	-	8	≥16	≤2	≤1	≤0.5
15	1	A	F	+	+	+	-	≥16	≥16	≤2	≥16	≤0.5
147	1	A	B	+	-	-	-	≥16	≥16	≤2	≥16	≤0.5
323	1	B	H	+	+	+	+	≥16	≥16	≤2	≥16	1.5
491	1	B	E	+	-	-	-	≥16	≥16	≤2	≥16	≤0.5

^*^A, ARNAS GH; B, AO VSC; C, AOUP

†MIC values were obtained by Etest

Among the resistance genetic determinants under screening, KPC3-type enzymes were detected in all *K*. *pneumoniae* isolates as a single determinant of β-lactam resistance (66 isolates, 70%) or in association with CTX-M-15 (28 isolates, 30%). Among the PMQR genes, *aac(6′)-Ib-cr* and *qnrB* were found in 73 (78%) and 34 (36%) isolates, respectively. Table 1 summarizes the main antibacterial drug resistance characteristics of the 94 isolates under study.

Bases upon a preliminary screening by comparing PFGE profiles, MLST of representative isolates recognized 10 different STs (Table 1). ST258 (37 isolates) or ST512 (one isolate) accounted for 40% of KPC-Kp isolates, followed by ST307 (27 isolates) accounting for 29% and ST273 (17 isolates) accounting for 18%. Additional six STs, 15, 101, 147, 323, 405 and 491 were identified in a number of isolates ranging between one and four (Table 1). The distribution of the three most prevalent STs showed they were all present in each of the three hospitals: in the ARNAS GH, AO VSC and AOUP, respectively, proportion of ST258 isolates was 34%, 53% and 44%, proportion of ST307 isolates was 28%, 47% and 12% and proportion of ST273 isolates was 21%, 12% and 13%.

Non susceptibility to aminoglycosides was unevenly distributed among the most common STs. Non susceptibility to amikacin was detected in 37 out of 38 (97%) ST258/512 isolates vs. none ST307 isolate and one out of 17 (6%) ST273 isolates. Conversely, non susceptibility to gentamicin was more frequently detected within ST307 and ST273 isolates compared with ST258/512 isolates: 19 isolates (70%) and all isolates (100%) vs. two isolates (5%). All the isolates belonging to the less prevalent STs proved to be susceptible to amikacin and non susceptible to gentamicin.

The proportion of isolates non susceptible to colistin was 42% (40 isolates) and ranged from 43% (16 isolates) for ST258 to 44% (12 isolates) for ST307 and 65% (11 isolates) for ST273. ST258 isolates had higher MIC values than those belonging to ST273 or ST307 (Table 1). All the isolates belonging to the less prevalent STs, except for one isolate belonging to ST323, were susceptible to colistin (Table 1).

Distribution of the genetic resistance determinants under study among the most prevalent STs was also uneven (Table 1). *bla*_CTX-M-15_ was present with a higher prevalence in ST307 (20 isolates, 74%), *aac-(6’)-Ib-cr* in 73 isolates (78%) with ST258 and ST307 isolates being 100% positive, and *qnrB* in 34 isolates (36%) with ST307 being 100% positive.

Table 2 summarizes the results of PCR and sequencing of the *mgrB* locus in relation with ST and colistin MIC values. The presence of an inactivation by insertion of an IS element was detected in all isolates attributed with ST273 and five out of 16 ST258 isolates. The presence of the non sense mutation t11a characterized 10 out of 12 colistin resistant ST307 isolates and only three ST28 isolates. Mutations leading to an aminoacid substitution were found in isolates belonging to STs 258 and 323. A total of eight isolates, six belonging to ST258 and two to ST307, carried a wild type *mgrB* gene (Table 2).

**Table 2:** Characteristics of the 40 colistin resistant KPC-Kp isolates described in this study.

ST	nr. of isolates	MICs of colistin (μg/mL)*	*mgrB* status†
	3	>256	t11a (non sense, premature termination)
258	3	16 - >256	insertional inactivation, IS*5*-like element at nt 75
	2	>256	t139c (W47R)
	2	>256	insertional inactivation, IS*1F*-like element at nt 105
	6	16 - >256	wild type
307	9	16–48	t11a (non sense, premature termination)
	2	3	wild type
	1	16	c64t (non sense, premature termination)
273	11	2–4	insertional inactivation, IS*1F*-like element at nt 61
323	1	1.5	g59t (W20L)

* MICs were obtained by Etest

† *mgrB* status was defined according with Cannatelli et al. [11]

## Discussion

Expansion of KPC-KP strains belonging to the hyperepidemic CC258 has been most responsible for the carbapenem non susceptible organisms epidemic in Italy [1]. A similar epidemiological picture has been as well described in several countries [19,20]. However, other widespread or local STs have been sporadically identified in high endemic areas, warning against a potential dissemination of *bla*_KPC_ genes among different Kp clones and enterobacterial species [20].

A countrywide survey on the diffusion of carbapenemases non susceptible Kp in Italy in the period May-June 2011 confirmed that most isolates belonged to CC258 (ST258 or ST512), while only a minority was ST101[1]. Moreover, production of KPC-type carbapenemases proved to be by far the most prevalent carbapenem resistance mechanism [1].

Accordingly, the epidemiology of KPC-Kp in Palermo, Italy, has been characterized by the emergence of KPC-Kp ST258 in the late 2008 [5] and then by the widespread acquisition of colistin resistance [9]. However, a recent preliminary investigation on a limited number of isolates suggested that a major epidemiological change was likely ongoing in our geographic area with ST258 being still prevalent, but circulating along with several additional STs, such as ST307, ST273 and ST323 [10]. This unexpected finding prompted us to conduct a more comprehensive six-month period of surveillance. Our present results confirm the predominance of KPC, but depict a radical change of the epidemiology of KPC-Kp which has moved from a substantially monoclonal circulation of ST258 towards a more complex polyclonal spread. Indeed, eight different non-CC258 STs have been recognized in the three largest acute general hospitals of Palermo, Italy. Of interest, the two most prevalent non-ST258 STs, e.g. ST307 and ST273, have been found in all three hospitals and in both ICU and non-ICU patients. This observation suggests a likely concurrent role of clonal expansion of some STs and lateral transmission of genetic resistance determinants.

A further issue of concern is the involvement in such a polyclonal epidemic not only of STs which have been previously recognized worldwide as carbapenemase producing international high risk clones, such as ST15 or ST101 [19,20], or have been identified also in Italy in *bla*_KPC_ carrying Kp strains, such as ST101 or ST147 [21], but also of less “reputed” STs, such as the more prevalent ST307 and ST273. Indeed, ST307 has been so far reported in a KPC-2 carrying strain clonally spreading in Texas [22] and as a major CTX-M producing clone in Pakistan [23]. Of interest, an intra-hospital dissemination of CTX-M-15 producing *K*. *pneumoniae* ST307, occurring in 2013 and involving a NICU, has been recently reported in Pavia, Italy [24]. ST273 has only recently been detected in Saint Petersburg, Russia, in KPC-2 producing *K*. *pneumoniae* [25]. Nevertheless, it has to be remembered that ST273 had been previously detected in one of our surveyed hospitals in colistin resistant, carbapenem susceptible, isolates which now have likely acquired *bla*_KPC_ through horizontal plasmid transmission [9].

Our epidemiological landscape appears to be unique in Europe. Indeed, in Spain and Norway multiple KPC-positive strains and plasmids have been identified in the last years in healthcare facilities giving rise to a complex molecular epidemiological picture [26,27]. However, in both these settings, described as low-endemic and low-frequency epidemic, respectively, ST258 was not previously established as the prominent clone in a long-term, high endemic scenario. Conversely, a parallel epidemiological evolution seems to emerge from a recent report on population structure of Kp from a tertiary care hospital in New York city, a high endemic area for KPC-Kp in the United States [28]. Indeed, among isolates from blood cultures recovered between January 2012 and July 2013, 10 STs were identified with ST258 still being predominant and accounting for 63% of KPC-Kp, ST17 and ST392 including several isolates and seven isolates being singletons [28]. Moreover, whilst in our hospitals, according with the Spanish experience, *bla*_KPC-3_ seems likely emerging in Kp lineages which have been already circulating as components of our local clonal pool, the very limited overlap between the STs of susceptible, cephalosporin resistant and KPC producing Kp isolates in the New York hospital jeopardizes inferences about progression in acquisition of resistance through plasmids uptake by previously resident isolates [28]. Further studies are needed to better understand the role of mobile resistance determinants in the polyclonal evolution of multidrug resistant Kp.

The widespread dissemination of resistance against colistin among KPC-Kp in Italy has been recently the subject of a national report by the European Survey on Carbapenemase-producing *Enterobacteriaceae* (EuSCAPE) Italian study group [4]. Our colistin resistance rate, quite similar to that reported by the survey, further underlines the size of the KPC-Kp crisis. The analysis of *mgrB* status shows that in our clinical setting alterations of the *mgrB* gene are strongly associated with the colistin resistant phenotype. Insertional inactivation of the *mgrB* gene was present in some ST258 isolates. This mechanism of alteration has been previously found by Cannatelli et al. [11] as the most common within a set of Italian and Greek KPC-Kp isolates. Insertional inactivation was also found in all ST273 isolates, whereas most colistin resistant ST307 isolates shared the non sense mutation t11a mechanism. This finding strongly suggests the role of cross-transmission within and between hospitals in our epidemiological setting. At our best knowledge this is the first report about *mgrB* status in a polyclonal KPC-Kp endemic setting.

Our study has some limitations. Epidemiological and clinical data about patients were not collected. Clonality was assessed by PFGE and MLST, but assessment of cross-transmission was not corroborated by conventional epidemiological data. Consequently, cross-transmission could have led to an overrepresentation of some pulsotypes/STs and some resistance patterns and mechanisms. Conversely, selection of non repetitive isolates could have underestimated the clonal diversity.

Our findings support the need to continuously monitor the KPC-Kp epidemiology and to assess by a nationwide survey the possible shifting towards a polyclonal epidemic. The peculiarities in the phenotypic and genetic resistance patterns of the different clones could be helpfully used in a wide scale screening approach.

Strategies of prevention and control of the KPC-Kp dissemination including a continuous surveillance system and the strict application of infection control measures are urgently necessary. These measures cannot be further postponed but are, unfortunately, more and more costly and demanding in a highly endemic situation like the one highlighted in Italy.
